# Uncoupling Molecular Testing for SARS-CoV-2 From International Supply Chains

**DOI:** 10.3389/fpubh.2021.808751

**Published:** 2022-01-24

**Authors:** Jo-Ann L. Stanton, Rory O'Brien, Richard J. Hall, Anastasia Chernyavtseva, Hye Jeong Ha, Lauren Jelley, Peter D. Mace, Alexander Klenov, Jackson M. Treece, John D. Fraser, Fiona Clow, Lewis Clarke, Yongdong Su, Harikrishnan M. Kurup, Vyacheslav V. Filichev, William Rolleston, Lee Law, Phillip M. Rendle, Lawrence D. Harris, James M. Wood, Thomas W. Scully, James E. Ussher, Jenny Grant, Timothy A. Hore, Tim V. Moser, Rhodri Harfoot, Blair Lawley, Miguel E. Quiñones-Mateu, Patrick Collins, Richard Blaikie

**Affiliations:** ^1^Department of Anatomy, School of Biomedical Sciences, University of Otago, Dunedin, New Zealand; ^2^MicroGEM NZ Ltd., Dunedin, New Zealand; ^3^Animal Health Laboratory, Ministry for Primary Industries—Manatu Ahu Matua, Upper Hutt, New Zealand; ^4^Clinical Virology, Institute of Environmental Science and Research Limited (ESR), Upper Hutt, New Zealand; ^5^Department of Biochemistry, School of Biomedical Sciences, University of Otago, Dunedin, New Zealand; ^6^Hudak Lab, Department of Biology, York University, Toronto, ON, Canada; ^7^Department of Molecular Medicine and Pathology, Faculty of Medical and Health Sciences, University of Auckland, Auckland, New Zealand; ^8^School of Fundamental Sciences, Massey University, Palmerston North, New Zealand; ^9^South Pacific Sera, Timaru, New Zealand; ^10^Ferrier Research Institute, Victoria University of Wellington, Lower Hutt, New Zealand; ^11^Department of Microbiology and Immunology, School of Biomedical Sciences, University of Otago, Dunedin, New Zealand; ^12^Molecular Pathology, Southern Community Laboratories, Dunedin, New Zealand; ^13^Research and Enterprise, University of Otago, Dunedin, New Zealand

**Keywords:** COVID-19, RT-qPCR, molecular reagents, supply chain, HomeBrew

## Abstract

The rapid global rise of COVID-19 from late 2019 caught major manufacturers of RT-qPCR reagents by surprise and threw into sharp focus the heavy reliance of molecular diagnostic providers on a handful of reagent suppliers. In addition, lockdown and transport bans, necessarily imposed to contain disease spread, put pressure on global supply lines with freight volumes severely restricted. These issues were acutely felt in New Zealand, an island nation located at the end of most supply lines. This led New Zealand scientists to pose the hypothetical question: in a doomsday scenario where access to COVID-19 RT-qPCR reagents became unavailable, would New Zealand possess the expertise and infrastructure to make its own reagents onshore? In this work we describe a review of New Zealand's COVID-19 test requirements, bring together local experts and resources to make all reagents for the RT-qPCR process, and create a COVID-19 diagnostic assay referred to as HomeBrew (HB) RT-qPCR from onshore synthesized components. This one-step RT-qPCR assay was evaluated using clinical samples and shown to be comparable to a commercial COVID-19 assay. Through this work we show New Zealand has both the expertise and, with sufficient lead time and forward planning, infrastructure capacity to meet reagent supply challenges if they were ever to emerge.

## Introduction

A stark lesson to emerge from the SARS-CoV-2 pandemic is an absolute reliance on global supply chains, centralized manufacture, and limiting production capacity for the specialized laboratory reagents required for contemporary diagnostic testing. This was clearly demonstrated in geographically-isolated New Zealand (NZ) early in the course of the COVID-19 pandemic by restricted availability of nasopharyngeal swabs, RNA extraction kits and the lag time evident from international suppliers to scale up molecular biology reagent and chemical production to meet rapidly increasing global demand. In the early stages of NZ's domestic pandemic response, it was estimated that there was as little as 5 days' reagent supply on hand to service rapidly escalating testing requirements. Any interruption to a global supply chain, itself experiencing unprecedented logistical challenges, could have had significant downstream consequences for disease response efforts applied at a local level. At the time, nations where reagents were manufactured also faced nationwide lockdowns in an attempt to contain the disease outbreak (1). Coupled with restricted reagent supply, issues affecting the importation of freight into a nation with closed borders further impacted the ability to source test reagents for necessary COVID-19 diagnostic testing and threatened ongoing security of supply. Significantly, NZ is separated from all other neighboring states by ocean, having no terrestrial borders. Australia, Fiji, New Caledonia and Tonga are its closest neighbors, with Australia being over 2,000 km away, across the Tasman Sea. This prompted the NZ science community to pose the question: in a doomsday scenario, just how many of the reagents required to perform a molecular COVID-19 screening test could be generated within NZ should the need arise?

The most widely used screening test for SARS-CoV-2 infection is reverse transcription polymerase chain reaction (RT-PCR) directed at targets in the SARS-CoV-2 RNA genome using fluorescent reporters in a real-time PCR format (1, 2). In this report, the abbreviation qPCR is used to denote real-time, fluorescence-mediated PCR, even in the absence of target quantitation, and RT-qPCR to denote reverse transcription qPCR as proposed by Bustin et al. (3). A sample is collected from the patient by nasopharyngeal swab, with the swab generally placed into a viral transport medium prior to processing. In high throughput diagnostic laboratories these swabs are processed to isolate RNA or total nucleic acid using automated processes such as the m2000 RealTime System (Abbott, Illinois, USA) or similar. Isolated nucleic acids are then used to detect the presence of SARS-CoV-2 RNA through nucleic acid amplification methods, most commonly RT-qPCR. A number of *in vitro* SARS-CoV-2 nucleic acid amplification tests have been approved by regulatory bodies for COVID-19 screening, such as TaqPath (Thermo Fisher Scientific), Alinity m SARS-CoV-2 assay (Abbott Molecular Inc.), and QIAstat-Dx Respiratory SARS-CoV-2 Panel (QIAGEN GmbH) (4). Many of these approved tests use the RT-qPCR amplification process to target and detect fragments of the SARS-CoV-2 genome. As a general rule, reagents required to perform reverse transcription and target sequence amplification by PCR are all sourced from large multinational companies with production usually based at central global locations, many either in the United States or in Europe.

Early in the pandemic a number of RT-qPCR assays were designed and published that could reliably detect viral RNA from nasopharyngeal swab samples with high sensitivity and specificity. Two of these, the E-gene target (5) and N gene target (6), have proven reliable and robust and have been incorporated into processing pipelines of some centralized laboratory facilities in NZ [for example, Southern Community Laboratories ((7), manuscript in preparation), and Environmental Science and Research Laboratories]. However, regardless of the target assay specifics, RT-qPCR diagnostics have the same basic and sequential steps in common: RNA extraction, reverse transcription and PCR. Within each step, specific sets of laboratory reagents are required to drive each process.

A team based out of NZ and the UK has developed and described an open platform for RNA extraction that utilizes magnetic beads for RNA capture. This protocol is referred to as BOMB.bio (www.bomb.bio/protocols) and all of the components for this extraction system can be readily made in a standard laboratory from off-the-shelf ingredients (8). With only minor modifications needed for it to be compatible with viral transfer medium from nasopharyngeal swabs, the BOMB.bio system was shown early in the pandemic to purify SARS-CoV-2 RNA in a high-throughput and clinically useful manner, with performance comparable to commercial kits (TP Jurkowski, TA Hore and KM Drake, *unpublished observations*).

Reverse transcription and PCR are performed sequentially either as two separate reactions or combined into a one-step mix where both processes occur sequentially within the same closed tube. A one-step approach is generally preferred as it is more efficient and requires less physical handling of the sample as there is no requirement to reopen the closed reaction tube to add or transfer PCR components. This thereby reduces the possibility of sample cross-contamination, the overall time to perform the test and is more suited to high-throughput laboratory workflows and commercial diagnostic applications. The two key enzymes driving each process are reverse transcriptase (RT), used to transcribe RNA into cDNA, and a thermostable DNA polymerase used to amplify target sequences of the SARS-CoV-2 genome to a detectable level. Other key reaction components include the deoxynucleoside triphosphate (dNTP) building blocks for making target DNA copies that arise from both RT and PCR reactions; the sequence-specific oligonucleotide primers necessary both for initiating cDNA synthesis and for delimiting the region of target amplification; and labeled oligonucleotide hydrolysis probes used to generate the fluorescent signal utilized by appropriate hardware to detect the accumulation of amplified target product. In addition, RNase inhibitor may be added to the RT reaction to help preserve RNA sample integrity.

In this work we describe the processes we used to engineer a RT-qPCR assay from NZ derived reagents. This includes making reverse transcriptase and thermostable DNA polymerase, purifying RNase inhibitor, and synthesizing dNTPs, oligonucleotide primers and dual-labeled hydrolysis probes from NZ-based, onshore resources. We tested our “Home Brewed” (HB) RT-qPCR system on SARS-CoV-2 RNA and clinical material demonstrating acceptable performance for COVID-19 screening in preparation for a worst case or “doomsday” scenario in which access to laboratory testing reagents from conventional sources is restricted or indeed eliminated entirely. This work assures ongoing and uninterrupted supply should conventional sources become compromised, if that were ever to eventuate.

## Methods

### Reverse Transcriptase Isolation and Testing

A plasmid encoding histidine-tagged thermostable reverse transcriptase (9) was kindly provided from https://pipettejockey.com. The plasmid was transformed into an expression host and purified in a conventional fashion. Briefly, the plasmid was transformed into *E. coli* strain BL21(DE3) and plated on LB agar plates supplemented with kanamycin (50 μg/ml). Resulting colonies were resuspended and inoculated into 1 L of LB media and grown at 37°C in a shaking incubator until an OD_600_ of 0.8 was reached. Transformants were then transferred to an 18°C incubator and grown for a further 50 min before being induced with isopropyl β-D-1-thiogalactopyranoside (0.2 mM final concentration) and cultured for an additional 14 h at 18°C.

The cell pellet was collected by centrifugation and resuspended in 35 ml of lysis buffer (50 mM Tris.HCL pH 8.0, 300 mM NaCl, 10% glycerol, 0.5% Triton X-100 and 10 mM imidazole) with the addition of 200 μl of lysozyme (25 mg/ml). Bacterial cells were lysed by sonication and the lysate clarified by centrifugation at 19,000 × *g* for 30 min. His-tagged protein was captured from the clarified lysate by batch binding on a rotator at 4°C for 30 min, using 1 ml of HIS-select resin (Sigma-Aldrich) previously equilibrated in lysis buffer. Resin was washed twice with 10 ml lysis buffer, followed by an additional two 10 ml washes with the same buffer incorporating 1 M NaCl to remove contaminating nucleic acids. The resin was then put into a gravity-flow column and transferred to buffer omitting Triton X-100 (50 mM Tris.HCL pH 8.0, 300 mM NaCl, 10% glycerol and 10 mM imidazole) with His-tagged protein eluted in the same buffer containing 300 mM imidazole. For storage, eluted protein was desalted into 50 mM Tris.HCL pH 8.0, 300 mM NaCl, 2 mM EDTA and 0.2% IGEPAL using an Econo-Pac® 10DG Desalting column (Bio-Rad), diluted two-fold with 100% glycerol and frozen at −20°C. As described, the protocol yielded a 6 ml solution of reverse transcriptase from 1 L of bacterial culture. Enzyme activity was determined using a modification of Vermeire et al. (10) substituting synthetic RNA for the SARS-CoV-2 E gene for MS2 RNA and using the SensiFast No-Rox probe RT-qPCR mix (Bioline) on a LightCycler 480 (Roche, Germany) to compare reverse transcriptase activity.

Reverse transcriptase purity was tested using a Mini-PROTEAN TGX Precast 8–16% gel with the Bio-Rad Mini PROTEAN Tetra System at 150 V for 30 min. The HYPERPAGE (Bioline) protein ladder was included to estimate size. Aliquots of reverse transcriptase were digested with prepGEM protease (MicroGEM, NZ) in 1 × Blue Buffer as per manufacturer's instructions and compared to undigested reverse transcriptase. Ten microliters of RT was combined with 9.5 μl Laemmli buffer (Bio-Rad) supplemented with 0.5 μl β-mercaptoethanol (Bio-Rad). The gel was washed three times with water then stained in Bio-Safe Coomassie stain (Bio-Rad) for 1 h with agitation. The gel was rinsed in water prior to viewing on a transilluminator.

### HB *Taq* Polymerase Expression, Purification and Activity

Thermostable DNA polymerase (*Taq* Pol I) from *Thermus aquaticus* was purified based on methods described by Chen et al. (11) and Pluthero (12). Briefly, an *E. coli* strain DH5α containing plasmid p*Taq* was grown in Terrific Broth (10 L total volume) with ampicillin selection (100 μg/ml). An overnight culture, 1/10 the final culture volume, was grown at 37°C with 200 rpm shaking and transferred to media and grown at 37°C, 200 rpm for 2 h. Polymerase expression was induced with 0.15 μM IPTG and the culture incubated at 37°C with 200 rpm shaking for 6–8 h. Bacteria were collected by centrifugation at 4,415 × *g* for 45 min and resuspended at ≤ 20% w/v in 20 mM Tris.HCL pH 8.5, 1 mM EDTA, 50 mM glucose and 1 mM PMSF. Resuspensions were frozen at −80°C and underwent three cycles of freeze/thaw. Bacterial cells were lysed using a multistep process; 100 μg/ml lysozyme was added to thawed resuspensions, mixed by inversion and incubated at room temperature for 15 min before 10 μg/ml each of DNAseI and RNAse was added and the lysates incubated at room temperature for 5 min. Suspensions were heated at 75°C for 30 min with inversion at 10 min intervals followed by sonication on ice at 50% power at 1 s pulses, for 1 min and subsequently 30 s. Cell debris was clarified by centrifugation at 16,000 × *g* for 10 min. The 75°C DNAaseI and RNAse treatment step was repeated and the supernatant further clarified by centrifugation. Polymerase protein was salted out by slowly adding a saturated ammonium sulphate solution while gently stirring on ice to a final 50% salt saturation. Precipitated *Taq* polymerase enzyme was collected by centrifugation 15,428 × *g* for 30 min, resuspended in 40 ml of a solution consisting of 20 mM Tris.HCL pH 8.5, 1 mM EDTA, 50 mM glucose, 1 mM PMSF, two Protease Inhibitor Cocktail tablets (Roche) and dialysed overnight in 20 mM Tris.HCL pH 8.5, 1 mM EDTA, 50 mM glucose for improved resuspension. Polymerase enzyme was further precipitated in 30% ammonium sulphate solution, collected by centrifugation and resuspended in 20 mM Tris.HCL pH 8.5, 1 mM EDTA and 50 mM glucose before being passed over a Phenyl Sepharose column (Pharmacia Biotech) equilibrated with 30% ammonium sulphate solution. The column was washed with 2 × column volumes (CV) 30% ammonium sulphate solution and *Taq* polymerase enzyme eluted with 5–10 CV of 10 mM HEPES pH 7.5 and 10% acetonitrile. Elution fractions containing *Taq* polymerase were dialysed in 20 mM HEPES pH 6.5 and purified over a MonoS 5/50 GL column (GE Healthcare), eluting with a 20 mM HEPES pH 6.5, 0.5 M KCl gradient. The final purified *Taq* polymerase fraction was dialysed in 20 mM HEPES pH 7.0, 50 mM KCl, 0.2 mM EDTA, 1% Tween-20, 1% NP40, filter sterilized and activity assessed. The volume was adjusted with buffer and an equal volume of glycerol added for storage at −20°C to give 5 U/μl and a final volume of 200 ml.

Polymerase activity was determined by comparing amplicon production between the purified HB *Taq* and a commercially-produced enzyme of known activity (*Taq* DNA Polymerase, recombinant, ThermoFisher Scientific, USA) using a conventional end-point PCR. Briefly, a pET32a-3C plasmid containing a cloned DNA fragment that generates a 659 bp amplicon when PCR amplified by forward and reverse plasmid primers was used as template. Five microliters of HB *Taq* or a comparable, commercial enzyme at variable dilutions was added to 15 μl 1 × PCR reaction mix (10 mM Tris HCL pH 9.0, 50 mM KCl, 0.1% v/v Triton-X100, 2.5 mM MgCl_2_, 0.2 mM of each dNTP, 0.5 μM of both forward and reverse primers, and 40 ng pDNA). Reactions were temperature cycled at 1 ×95°C for 3 min followed by 25 cycles at 95°C for 30 s, 60°C for 1 min and a final 1 ×72°C for 5 min. Following amplification 10 μl of each PCR reaction was electrophoresed on a 1% agarose gel stained with SybrSafe (Thermo Fisher Scientific, USA). Agarose gels were imaged using a ChemiDoc Gel Imaging system (BioRad) and the Image Lab software (BioRad) used to determine relative fluorescent band intensity.

### RNase Inhibitor

RNase inhibitor was purified from sheep liver following the protocol described by Garcia and Klebe (13). Briefly, fresh liver was homogenized with a food blender in equal weight of 0.25 M sucrose, 0.1 M potassium phosphate (pH 7.5), 5 mM mercaptoethanol, 5 mM DTT and 1 mM EDTA. DTT was added fresh into the buffer on the day of use. The homogenate was centrifuged (4,122 × *g* for 30 min), the supernatant decanted and the pH adjusted to 7.2 using 2.5 M KOH. For every 100 ml of supernatant, 80 ml of 50% (w/v) PEG-3350, 0.05 M potassium phosphate (pH 7.2) was slowly added to a final concentration of 22.2%. The supernatant was centrifuged (4,122 × *g* for 90 min), supernatant poured through cheesecloth and the pH adjusted to 4.35–4.40 using glacial acetic acid. The supernatant was centrifuged (4,122 × *g* for 90 min) and the pellet resuspended with 0.1 M potassium phosphate (pH 7.2), 5 mM DTT and 1 mM EDTA. The resuspended pellet was centrifuged again (4,122 × *g* for 20 min) and the supernatant kept as an isoelectric precipitate. The remaining pellet was resuspended again, centrifuged (4,122 × *g* for 20 min) and the supernatant pooled with isoelectric precipitation.

Ten milligrams of RNase A (Sigma R6513) was conjugated to 1 g of CnBr activated-Sepharose 4B (17043001) as per Cytiva/GE instruction 71-7086-00 AF. RNaseA-Sepharose beads were added to the isoelectric precipitate with binding at room temperature for 1 h. The RNaseA-Sepharose beads were collected either through a Buchner funnel or by centrifugation in a 50 ml tube at 20 × *g* for 5 min and packed onto a BioRad Econo column (1 ×20 cm). Isoelectric precipitate was removed first, the RNaseA-Sepharose transferred into the column and packed under gravity flow. The affinity column was washed with 0.1 M potassium phosphate (pH 6.4), 0.5 M NaCl, 5 mM DTT and 1 mM EDTA buffer (14). The RNase inhibitor was eluted with 0.1 M borate (pH 6.4), 4.0 M NaCl and 15% (v/v) glycerol and collected in 1 ml fractions at a flow rate of 0.5–1 ml/min. The fractions with RNase inhibitor activity were identified, pooled and dialysed overnight with 20 mM HEPES (pH 7.6), 50 mM KCl, 8 mM DTT and 50% (v/v) glycerol storage buffer. RNase inhibitor was concentrated to 40 U/μl using an Amicon ultra-15 10 KDa ultrafiltration device (Millipore) and stored at −20°C. RNase inhibitor assay was performed as Garcia and Klebe (13) described with the exception that uranyl acetate was replaced with 0.5% phosphotungstic acid.

### Oligonucleotide Synthesis and Purification

Oligonucleotides (ONs) were synthesized using a Mermaid-4 automated DNA synthesizer (BioAutomation Corp., USA) using 5-ethylthio-1*H*-tetrazole as an activator. Oxidation and deprotection times were 80 s (repeated three times) and coupling time was 120 s for a 5 μmol synthesis scale. The resulting ONs were cleaved from the solid support and deprotected with concentrated aqueous ammonia (~28%) at room temperature for 2 h followed by 55°C for 12 h for DNA primers, and at room temperature for 24 h for DNA probes.

For primers, purification of ONs was accomplished by ion-exchange (IE) HPLC using an IE-column (TSKgel Super Q-5PW) with Buffer A (20 mM Tris-HCl, 1 mM Na_2_-EDTA, pH 9.0), and Buffer B (20 mM Tris-HCl, 1 mM Na_2_-EDTA, 1M NaCl, pH 9.0). Gradients were 3.7 min 100% A, convex curve gradient to 40% B in 11.1 min, linear gradient to 100% B in 43.9 min, kept at 100% B for 10 min and then change to 100% A over 5 min. Collected individual UV-absorbing fractions (λ = 260 nm) were further purified by reverse-phase (RP) HPLC using an RP-column (Hypersil GOLD^TM^ from Thermo Fischer Scientific) and Buffer C (100 mM aq. triethylammonium acetate, pH 7.0) and Buffer D (acetonitrile). Gradients were 2 min 100% C, linear gradient to 25% D in 18 min, linear gradient to 80% D over 1 min, linear gradient to 100% D over 7 min. Collected individual UV-absorbing fractions (λ = 260 nm) were desalted using a NAP-25 column (Amersham Biosciences). The composition of each fraction was confirmed by ESI-MS, and the desired ONs were identified and lyophilized.

For probes containing fluorescein and BHQ-1 at 5′- and 3′- ends, respectively (see Table 1 for sequences and **Figure 4** for reagents used for the DNA synthesis) purification was accomplished using preparative denaturing 20% polyacrylamide gel electrophoresis (PAGE). Gels were prepared in 1 × TBE buffer (7 M urea, pH 8.0) with 2 mm thickness, 17.5 ×14.5 cm^2^ (19:1 acrylamide/bis-acrylamide ratio). Samples (200 μl) were mixed with 7 M urea (250 μl) and incubated at 90°C for 5 min to disrupt higher order assemblies. 1 × TBE buffer (pH 8.0) was used as a running buffer. All gel electrophoresis was performed at 5°C in the dark to avoid photodegradation of fluorophores. After the electrophoresis, a band corresponding to the desired product was cut from the gel. Labeled ON was eluted from the gel using an electroelution device (Genterra, Russia; 290 V, 150 mA, 43.5 W) allowing DNA to be concentrated and collected in a trap made of a large pore size membrane and a dense, inert, non-absorbent membrane. Electroeluted DNA was further purified by reverse phase HPLC as mentioned above. Collected individual UV-absorbing fractions (λ = 260 nm) were desalted using a NAP 25 column. The composition of probes was confirmed by ESI-MS (Table 1), and the purity of the products was found to be at least 80% as determined by denaturing PAGE (7 M urea). Gels were visualized using a Fujifilm FLA-5000 imaging system (532 nm, LPG channel to detect fluorescein and BHQ-1 containing ONs), then stained with Stains All (Sigma Aldrich), and imaged using GelDoc (Bio-Rad) after destaining in water.

**Table 1:** List of DNA primers and probes synthesized.

	**Name**	**DNA sequence, 5^**′**^-3^**′**^**	**MW (g/mole)**		**Molar extinction coefficient (L/mole/cm)**	**Amount obtained (nmol)**	**Retention time (min)**		**Yield (%)** ^**c**^
			**Calculated**	**Observed**			**IE^**a**^**	**RP** ^**b**^	
E_Sarbeco	F (CV1)	ACAGGTACGTTAATAGTTAATAGCGT	8,033.3	8,032.3	269,500	102.4	23.0	15.4	1.0^d^
	R (CV2)	ATATTGCAGCAGTACGCACACA	6,712.4	6,712.1	221,000	403.6	24.0	15.6	4.0^d^
N_Sarbeco	F (CV3)	CACATTGGCACCCGCAATC	5,717.8	5,717.3	174,200	220.4	21.1	15.0	2.2^d^
	R (CV4)	GAGGAACGAGAAGAGGCTTG	6,280.1	6,279.0	211,300	352.2	20.0	14.6	3.5^d^
CDC RNP3	F (Control-1)	CCAAGTGTGAGGGCTGAAAAG	6,544.3	6,543.1	214,600	699	20.2	14.5	7.0^d^
	R (Control-2)	TGTTGTGGCTGATGAACTATAAAAGG	8,089.3	8,089.3	262,200	393.6	22.7	15.1	3.9^d^
CDC N gene Primers	Forward	GGGGAACTTCTCCTGCTAGAAT	6,685.4	6,684.1	202,400	1,100	22.6	14.0	11^d^
	Reverse	CAGACATTTTGCTCTCAAGCTG	6,750.4	6,750.1	208,600	1,640	22.3	13.8	16.4^d^
N-Gene probe	CDC N-Gene	/FAM/TTGCTGCTGCTTGACAGATT/BHQ-1/^f^	7,206.0	7,206.2	208,860	22.8	–^g^	23.9	0.46^e^
E-Gene probe	E-Sarbeco-P1	/FAM/ACACTAGCCATCCTTACTGCGCTTCG/BHQ-1/	8,934.1	8,933.5	262,360	17.2	–	23.9	0.34^e^
Control probe	CDC RNP3 probe	/FAM/CCCCAGTCTCTGTCAGCACTCCCTTC/BHQ-1/	8,846.0	8,846.5	248,960	12	–	23.9	0.24^e^

a *IE, ion-exchange HPLC, conditions are described above*.

b *RP, reverse-phase HPLC, conditions are described above*.

c *Overall yield is calculated based on the ratio of amount of isolated and purified DNA over loading of the first nucleotide on a solid support*.

d *Synthesized at 5 μmol scale, two columns*.

e *Synthesized at 5 μmol scale, one column*.

f */FAM/ stands for Fluorescein, 6-isomer (from Lumiprobe, Catalog number: C5160; CAS number: 204697-37-0); /BHQ-1/ stands for Black Hole Quencher^®^-1 [from Genterra (Russia), Cat. No.: OR-Q-002-3-5A-1000]*.

g *Purified by 20% denaturing PAGE*.

### dNTP Synthesis

2′-Deoxycytidine hydrochloride salt (Chem-Impex Int'l. Inc.) and thymidine (AK Scientific, Inc.), 2′-deoxyadenosine (Biosynth Carbosynth) and 2′-deoxyguanosine monohydrate (Biosynth Carbosynth) were sourced from the specified vendors and used as received. Other reagents and solvents used in the synthesis were sourced from Sigma Aldrich (imidazole; triethylamine; trifluoroacetic acid; phosphoryl chloride; sodium pyrophosphate tetrabasic decahydrate; tributylamine; trimethyl phosphate; Dowex® 50W X8 hydrogen form, strongly acidic, 200–400-mesh; tetrahydrofuran; dimethylformamide; acetonitrile; pyridine, AK Scientific, Inc. (proton sponge) and Biosynth Carbosynth [*tert*-butyldimethylsilyl (TBDMS) chloride]. Phosphoryl chloride was freshly distilled and stored under argon according to the method of Williams and Harris (15), while trimethyl phosphate was stored over activated 3 Å molecular sieves under argon for at least 48 h prior to use.

3′-O-TBDMS-protected deoxynucleosides were each prepared on a gram-scale following described methods. Briefly, the deoxynucleosides were treated with excess TBDMS chloride and imidazole following the method of Grover et al. (16), resulting in silylation of both the 3′- and 5′-alcohols. The bis-silyl ethers thus obtained were subjected to trifluoroacetic acid-mediated hydrolysis of the 5′-silyl ether following the method of Zhu et al. (17), providing the 3′-O-TBDMS-protected deoxynucleosides.

The 3′-O-TBDMS-protected deoxynucleosides were then used to prepare the dNTPs by a sequence consisting of 5′-triphosphorylation, purification by reverse phase flash chromatography, acid-mediated silyl ether cleavage, then conversion of the dNTPs to their sodium salt forms. Reaction progress could be monitored at each step by thin layer chromatography on silica gel using *i*-PrOH-H_2_O-conc aq. NH_4_OH (6:1:3, or 5:2:3) as eluent, with visualization under UV lamp at 254 nm or KMnO_4_ stain.

General procedure: to a solution of 3′-*O*-TBDMS-deoxynucleoside and additive (1.5 mol equiv. proton sponge for 3′-*O*-TBDMS-dT; 2.0 mol equiv. tributylamine for 3′-*O*-TBDMS-dA; no additive for 3′-*O*-TBDMS-dC or 3′-*O*-TBDMS-dG) in anhydrous trimethyl phosphate (0.25 M) at 0°C was added a solution of phosphoryl chloride (2 M in trimethyl phosphate, 1.1 mol equiv.) dropwise. The reaction mixture was stirred at this temperature for 30 min, then treated with tributylamine (4.0 mol equiv.) and a solution of bis(tributylammonium) pyrophosphate (0.5 M in acetonitrile, 2.0 mol equiv.) (15). The reaction mixture was stirred at 0°C for a further 30 min, then quenched by addition of excess 1 M aq. triethylammonium bicarbonate buffer (pH 8.5). The reaction mixture was diluted with deionised water, then washed with dichloromethane three times to remove organic-soluble impurities. The aqueous layer was concentrated *in vacuo* at or below 30°C until most volatiles were removed, then the crude oil was purified by reverse-phase flash chromatography on a C18 cartridge (Buchi FlashPure ID, 40 μm, irregular particle size) using a Buchi Pure automated chromatography system. The product was eluted with three CV of 5% MeOH in H_2_O containing 1% triethylamine, followed by a gradient of 5–30% MeOH in H_2_O containing 1% triethylamine over 15 CV. Product-containing fractions were partially concentrated *in vacuo* to remove MeOH, then lyophilized to afford the purified 3′-O-TBDMS-dNTPs. 3′-O-TBDMS-dNTPs were then dissolved in deionised water (0.05 M) and treated with MeOH-washed Dowex® 50W X8 hydrogen form resin (1 g/g of 3′-O-TBDMS-dNTP). This suspension was stirred at room temperature for 1 h, then neutralized by addition of 1 M aq. triethylammonium bicarbonate buffer (pH 8.5). The mixture was filtered to remove the resin, and the resin was washed twice with a small quantity of deionised water. The filtrate was lyophilized to afford dNTPs as their tris(triethylammonium) salts. Ion exchange through Dowex® 50W X8 Na-form resin, followed by lyophilization afforded dNTPs as their sodium salts. The dNTPs were characterized by NMR and high resolution ESI-MS, and assessed for purity by reverse phase HPLC using UV peak area at 260 nm: 1.0 μl of a 1 mg/ml aqueous solution of each dNTP was injected onto an Agilent Poroshell 120 EC-C18, 2.7 μM, 100 ×4.6 mm column and eluted with a linear gradient of 0–15% MeCN in 50 mM aqueous triethylammonium acetate (pH 7.0) with 2 mM EDTA over 10 min at a flow rate of 1.0 ml min^−1^.

2′-Deoxycytidine-5′-triphosphate sodium salt (50.5 mg, 37% yield from 3′-O-TBDMS-dC, 90.8% purity): ^**1**^**H NMR** (500 MHz, D_2_O) δ 8.02 (d, *J* = 7.6 Hz, 1H), 6.38 (t, *J* = 6.6 Hz, 1H), 6.20 (d, *J* = 7.6 Hz, 1H), 4.70–4.65 (m, 1H), 4.27 (d, *J* = 4.0 Hz, 3H), 2.52–2.33 (m, 2H); ^**13**^**C NMR** (126 MHz, D_2_O) δ 165.8, 157.0, 141.8, 96.5, 86.0, 85.5 (d, ^3^*J*_CP_ = 9.1 Hz), 70.7, 65.3 (d, ^2^*J*_CP_ = 5.7 Hz), 39.4; ^**31**^**P NMR** (202 MHz, D_2_O) δ −9.9 (d, *J*_PP_ = 19.3 Hz), −11.2 (d, *J*_PP_ = 19.3 Hz), −22.6 (t, *J*_PP_ = 19.6 Hz); **HRMS** (ESI–): Calculated for: C_9_H_15_N_3_O_13_P_3_ 465.9818. Found [M – H]^−^: 465.9826.

Thymidine-5′-triphosphate sodium salt (1.26 g, 54% yield from 3′-O-TBDMS-dT, 95.7% purity): ^**1**^**H NMR** (500 MHz, D_2_O) δ 7.80 (s, 1H), 6.41 (t, *J* = 6.9 Hz, 1H), 4.74–4.70 (m, 1H), 4.33–4.20 (m, 3H), 2.49–2.38 (m, 2H), 1.99 (s, 3H); ^**13**^**C NMR** (126 MHz, D_2_O) δ 166.6, 151.8, 137.4, 111.8, 85.5 (d, ^3^*J*_CP_ = 9.5 Hz), 85.0, 70.9, 65.5 (d, ^2^*J*_CP_ = 5.7 Hz), 38.6, 11.7; ^**31**^**P NMR** (202 MHz, D_2_O) δ −10.3 (d, *J*_PP_ = 19.3 Hz), −11.4 (d, *J*_PP_ = 19.9 Hz), −22.8 (t, *J*_PP_ = 19.6 Hz); **HRMS** (ESI–): Calculated for: C_10_H_16_N_2_O_14_P_3_ 480.9814. Found [M – H]^−^: 480.9821.

2′-Deoxyguanosine-5′-triphosphate sodium salt (54.6 mg, 38% yield from 3′-O-TBDMS-dG, 94.5% purity): ^**1**^**H NMR** (500 MHz, D_2_O) δ 8.14 (s, 1H), 6.34 (t, *J* = 6.9 Hz, 1H), 4.83 (dt, *J* = 6.4, 3.5 Hz, 1H), 4.34–4.30 (m, 1H), 4.29–4.19 (m, 2H), 2.87–2.53 (m, 2H); ^**13**^**C NMR** (126 MHz, D_2_O) δ 158.9, 153.8, 151.3, 137.6, 116.2, 85.7 (d, ^3^*J*_CP_ = 9.1 Hz), 83.6, 71.1, 65.5 (d, ^2^*J*_CP_ = 5.7 Hz), 38.7, 38.6; ^**31**^**P NMR** (202 MHz, D_2_O) δ −8.85 (d, *J*_PP_ = 19.3 Hz), −10.99 (d, *J*_PP_ = 19.3 Hz), −22.29 (t, *J*_PP_ = 19.3 Hz); **HRMS** (ESI–): Calculated for: C_10_H_15_N_5_O_13_P_3_ 505.9879. Found [M – H]^−^: 505.9884.

2′-Deoxyadenosine-5′-triphosphate sodium salt (54.2 mg, 41% yield from 3′-O-TBDMS-dA, 94.5% purity): ^**1**^**H NMR** (500 MHz, D_2_O) δ 8.50 (s, 1H), 8.25 (s, 1H), 6.53 (t, *J* = 6.8 Hz, 1H), 4.85 (dt, *J* = 6.6, 3.4 Hz, 1H), 4.38–4.33 (m, 1H), 4.31–4.19 (m, 2H), 2.90–2.62 (m, 2H); ^**13**^**C NMR** (126 MHz, D_2_O) δ 155.4, 152.5, 148.6, 140.0, 118.6, 85.8 (d, ^3^*J*_CP_ = 9.1 Hz), 83.7, 71.1, 65.5 (d, ^2^*J*_CP_ = 5.7 Hz), 39.1; ^**31**^**P NMR** (202 MHz, D_2_O) δ −9.7 (d, *J*_PP_ = 19.3 Hz), −11.1 (d, *J*_PP_ = 19.9 Hz), −22.5 (t, *J*_PP_ = 19.9 Hz); **HRMS** (ESI–): Calculated for: C_10_H_15_N_5_O_12_P_3_ 489.9930. Found [M – H]^−^: 489.9935.

### SARS-CoV-2 Reference Materials: SARS-CoV-2 Reference RNA + Synthetic E Gene

Reference RNA for this work was prepared by the Viral Pathogenesis Laboratory, Microbiology and Immunology Department, University of Otago, using a sample obtained from an infected patient in Dunedin, NZ (7). Briefly, the positive clinical specimen was inoculated into VERO cells and incubated for 3–7 days at 37°C, with 5% CO_2_. Culture material was inactivated using the Zymo ZR Viral RNA Kit™ (catalog number: R1035) and the RNA stored at −80°C. In addition, the Institute of Environmental Science and Research (ESR) laboratory has now successfully grown over 80 SARS-CoV-2 isolates for research with three of these isolates grown in substantial amounts to serve as reference material for NZ researchers.

A synthetic E-gene reference RNA was made for this work. A genome region downstream of the ORF3a gene stop codon, through the E gene, to the M gene start codon (302 bp) was synthetically generated and cloned into a pBluescript II KS(+) vector by Genscript (Piscataway, NJ, USA). RNA template was generated after linearising the plasmid by *Xho*I digest and subsequent *in vitro* transcription, from the T7 promoter, using the Invitrogen Maxiscript *in-vitro* transcription kit (ThermoFisher) according to the manufacturer's instructions. RNA quantity was measured using the Qubit RNA HS Assay kit (ThermoFisher).

### HB RT-PCR Protocol

Assessment of RT-qPCR performance of the prepared reagents was carried out using a 7500 Fast Real-Time PCR instrument (Applied Biosystems); one-step RT-qPCR cycling conditions comprised an initial reverse transcription step at 50°C for 20 min followed by a denaturation step at 95°C for 5 min and 40 cycles of qPCR comprising 95°C for 15 s and 60°C for 1 min.

Two RT-qPCR mix preparations were taken forward for clinical evaluation and validation on COVID-19 patient samples. A 2 × RT-qPCR reaction mix, excluding the enzymatic components, was prepared accordingly, consisting of 100 mM Tris.HCl (pH 8.4), 150 mM KCl, 5 mM MgCl_2_, 1.6 μM forward primer, 1.6 μM reverse primer, 1.2 μM hydrolysis probe, 0.8 mM dNTPs, and 50 ng/μl DNA. Similarly, a 10 × enzyme mix solution was prepared comprising 50% RT enzyme preparation and *Taq* enzyme preparation at ~0.5 U/μl, prepared in enzyme storage buffer (10 mM HEPES pH 7.0, 25 mM KCl, 0.1 mM EDTA, 1 mM DTT, 0.5% v/v Tween-20, 0.5% NP40, 50% glycerol).

## Results

### The Scale of the Challenge

**Figure 1:**
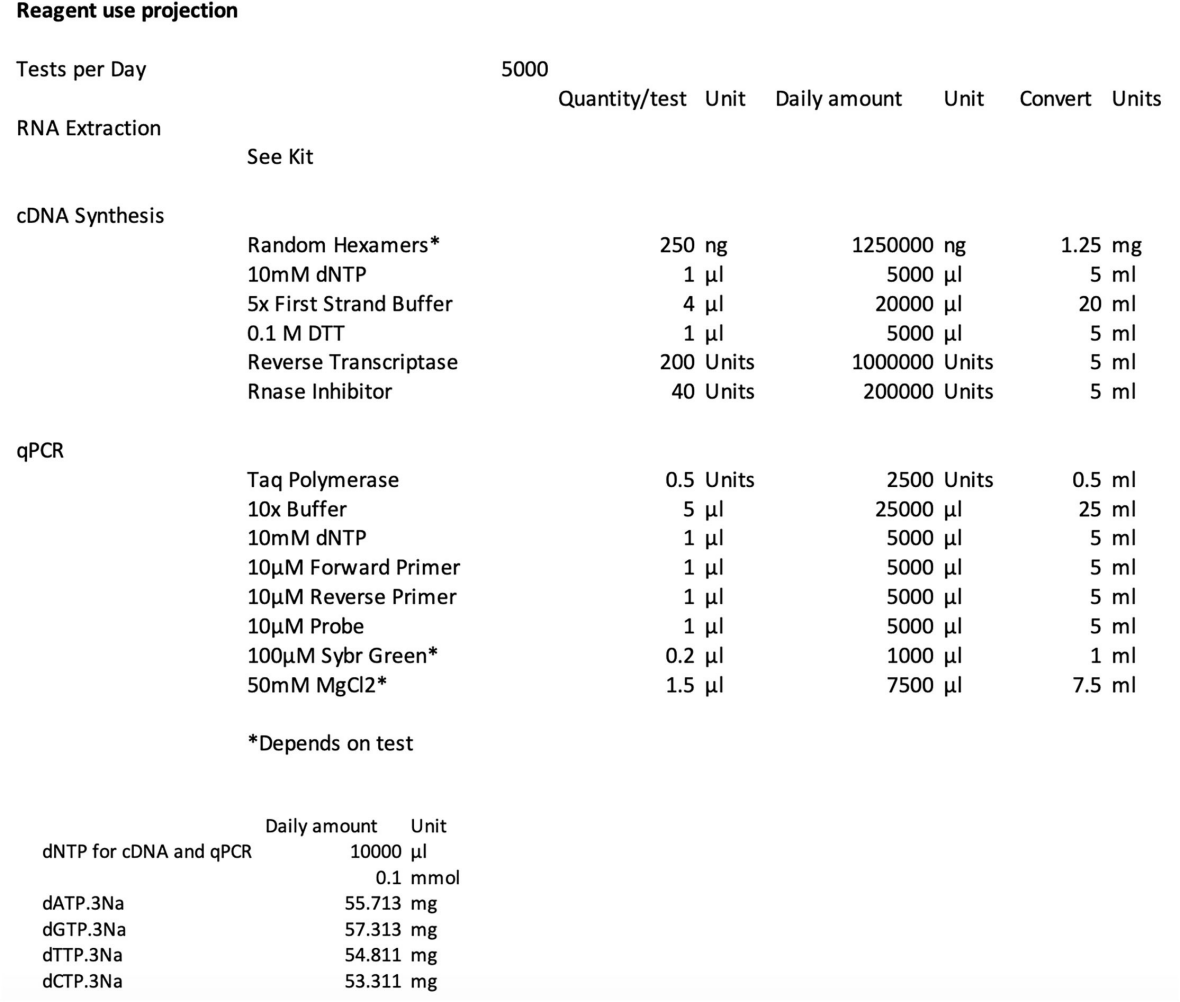
Interactive reagent calculator used to determine what, and in what quantity, reagents were required for a successful onshore production scheme. Not all components used in this reagent calculator contributed to the final HB RT-qPCR assay.

The first step when contemplating NZ's ability to produce sufficient reagent onshore to service COVID-19 screening needs was to identify the critical, individual components necessary for RT-qPCR testing and to estimate the likely daily quantities that might be required to meet demand. A simple interactive reagent calculator was created modeled on a commercial two-step RT-qPCR (Figure 1). Test number was assigned to an average of 5,000 tests per day, the projected level of required daily testing estimated during the early phase of NZ's pandemic response. This suggested that target quantities for individual components could be achieved with the current production infrastructure.

In addition to scoping the size of the manufacturing challenge, this calculator identified the individual components that needed to be prepared if self-sufficiency goals were to be realized. Reagents were divided into two categories: (i) general items that could be made by any company producing biologically-based products and (ii) more specialized reagents. These specialized reagents included the thermostable *Taq* DNA polymerase, reverse transcriptase enzyme, dNTPs, RNase inhibitor and the oligonucleotides necessary for the RT-qPCR assay. In addition, hydrolysis probe-based RT-qPCR was considered the preferred option as it would most likely provide higher specificity when compared to DNA binding dye detection-based chemistry (such as SYBR Green) and preclude post-PCR manipulations or inspection such as melt-curve analysis. We elected to use SARS-CoV-2-specific primers for priming reverse transcription however it was also possible to synthesize random hexamers onshore if required.

### dNTP Synthesis

**Figure 2:**
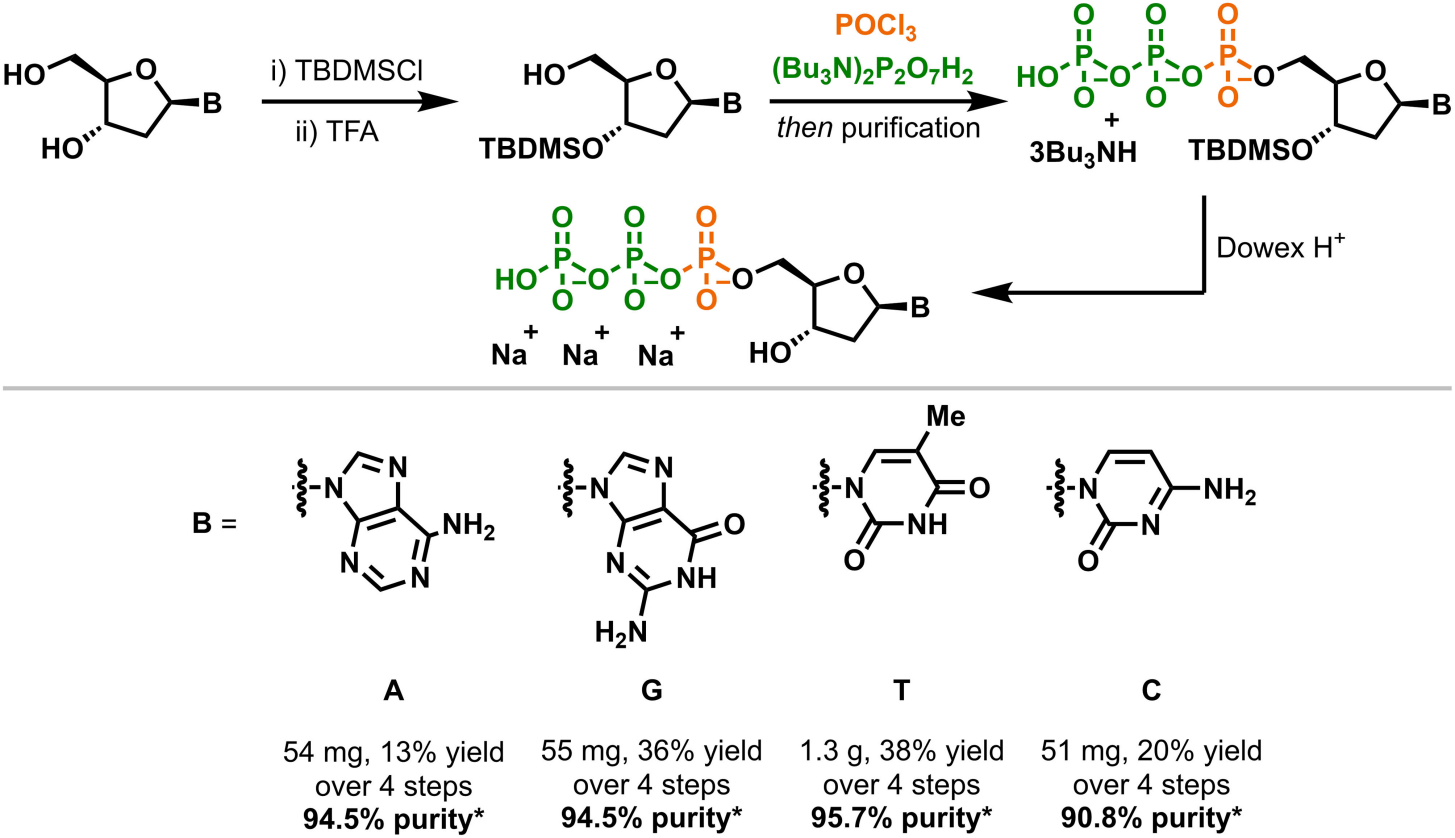
Overview of the synthesis of the dNTPs. *Purity was determined by UV peak area at 260 nm by reverse phase HPLC (details in text).

Following methodology recently developed in our laboratories (18), the 2′-deoxynucleoside 5′-triphosphates (dNTPs) were prepared using a novel chemical synthesis from their respective 2′-deoxynucleosides, that are readily available from overseas commercial vendors (Figure 2). While dNTPs can be prepared directly from deoxynucleosides (19), purification of the dNTPs prepared by this method is challenging on a large scale. Instead, the 3′-alcohol of each deoxynucleoside was protected as a tert-butyldimethylsilyl (TBDMS) ether prior to triphosphorylation and this lipophilic group improved purification by reverse phase flash chromatography. The TBDMS group could then be cleaved under mildly acidic conditions to furnish the dNTPs cleanly. For this work we purchased 100 g of each 2′-deoxynucleoside at a cost of approximately one USD/g (notably, kilogram quantities of these compounds are usually available from the vendor; Carbosynth, Compton, UK). For comparison, the same vendor currently offers three of the four 2′-deoxynucleoside triphosphates at 500–750 USD/g and have only 1–4 g total stock of each dNTP. The other reagents necessary for this synthesis are widely available and, with only a few exceptions, can be found in a typical organic synthesis laboratory. While numerous phosphorus sources can be used to prepare triphosphates, we opted to construct the dNTPs from sodium pyrophosphate (Na_4_P_2_O_7_) and phosphorus oxychloride (POCl_3_). It is worth noting that POCl_3_ is not permitted for transport by airfreight for safety reasons and must therefore be imported by sea freight, which presents a possible bottleneck in dNTP production. Fortunately, POCl_3_ can be purchased in large quantities and is stable indefinitely when stored correctly.

Yields for the preparation of the four dNTPs ranged from 13 to 38%, however it is anticipated these could be increased with further optimisation of the synthetic method. While only milligram quantities of each dNTP were required, 1.3 g of dTTP was synthesized to demonstrate the method could be scaled up as necessary.

**Figure 3:**
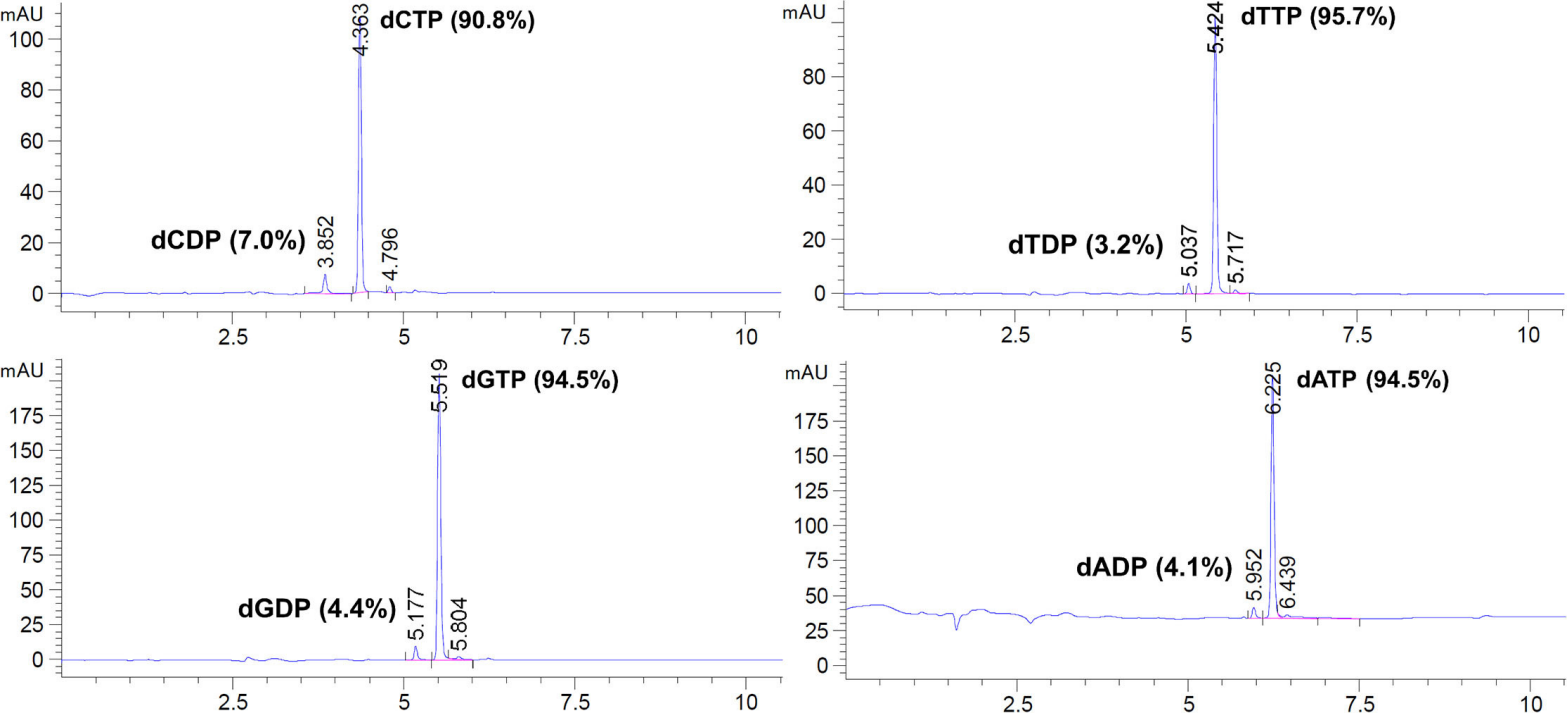
dNTP purity analysis by HPLC UV peak area at 260 nm. Method: C18 column (Agilent Poroshell 120 EC-C18, 2.7 μM, 100 ×4.6 mm), linear gradient of 0–15% MeCN in 50 mM aqueous triethylammonium acetate (pH 7.0) with 2 mM EDTA over 10 min at a flow rate of 1.0 ml min^−1^. The retention times of the peaks (in minutes) are shown on the x axes, and the peak intensities in milli-absorbance units (mAU) at 260 nm are shown on the y axes.

Commercially sourced dNTPs are usually prepared to 99.0% or higher purity, which is quantified by reverse phase HPLC UV peak area. We therefore developed HPLC conditions for the analysis of the dNTPs prepared in this work and found their purity ranged from 90.8 to 95.7% (Figure 3). In each case, a major impurity with shorter retention time than the dNTP was observed. Further analysis by liquid chromatography mass spectrometry (LCMS) determined that these impurities were the respective 2′-deoxynucleoside 5′-diphosphates (dNDPs). These dNDPs, which constitute a 3–7% impurity in the dNTPs, might form as a result of either impurities in the pyrophosphate reagent used in this synthesis, or by decomposition of the dNTPs during the later stages of the synthesis. Impurities with longer retention times than the dNTPs were also observed, constituting 0.8–2.2% of each product, however the identity of these impurities could not be determined.

### Synthesis of DNA Primers and Probes

**Figure 4:**
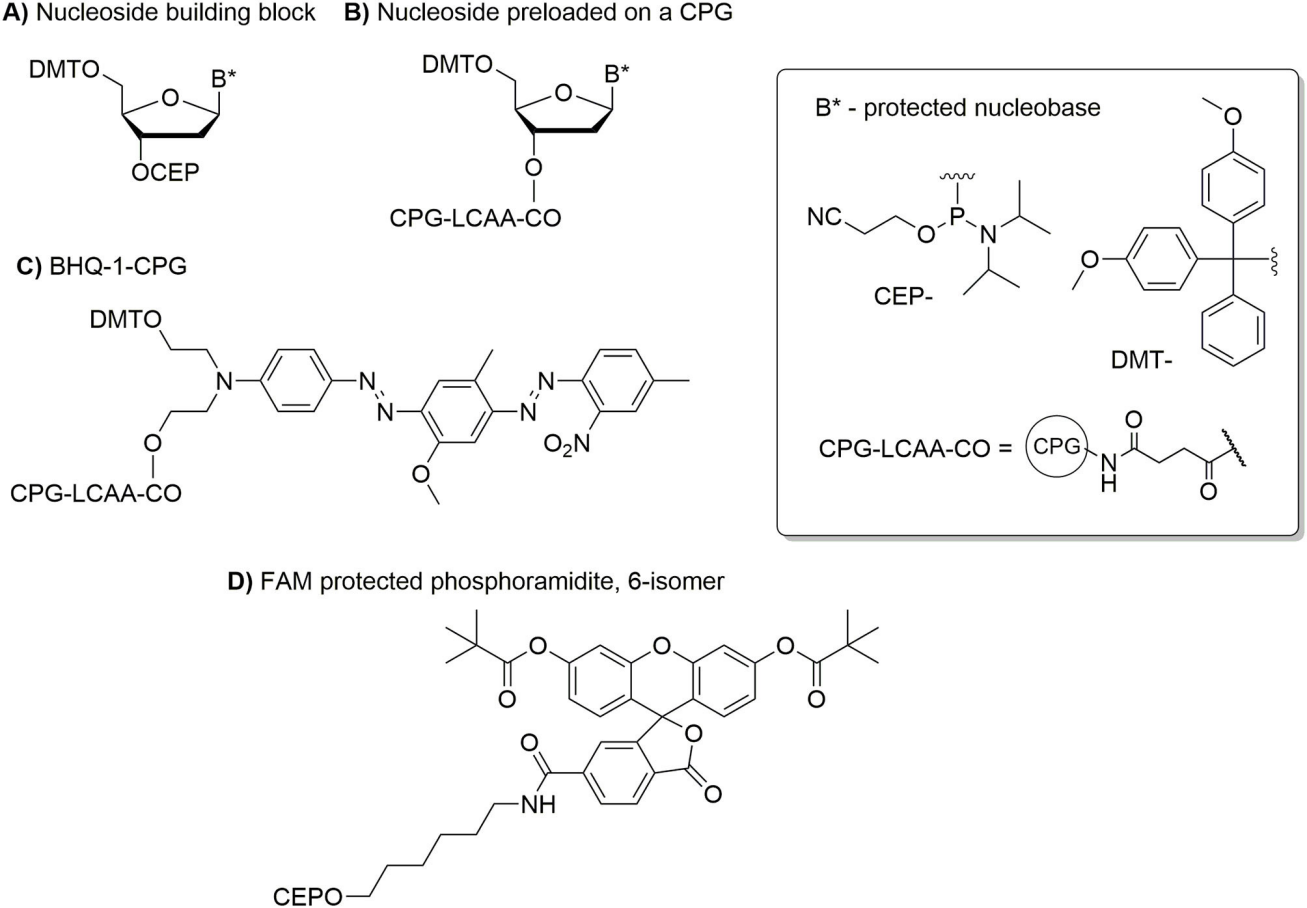
DNA monomers used for the synthesis of DNA primers and probes. **(A)** Structure of a 5′-*O*-DMT protected nucleoside phosphoramidite used for an automated DNA synthesis. **(B)** Structure of a 5′-*O*-DMT protected nucleoside bound to the CPG-support and used as the first nucleotide at the 3′-end of the DNA sequence. **(C)** Structure of a DMT-protected BHQ-1 bound to the CPG support and used for the synthesis of DNA probes with BHQ-1 present at 3′-end. **(D)** Structure of fluorescein containing phosphoramidite used for installation of fluorescein at the 5'end of the DNA probe.

Figure 4 shows the specialized nucleotide reagents used for the synthesis of DNA primers and probes. Additional DNA phosphoramidites, supports and labeling reagents are required and these were obtained from overseas suppliers. However, these reagents also can be synthesized from standard chemicals, that are usually available in bulk, using established and published procedures [e.g., (20)]. If all reagents are available, the synthesis of DNA primers and probes can be accomplished in 1–2 days followed by 2–4 days required for thorough purification and isolation of the products.

Automated DNA synthesis was used for preparation of DNA primers and probes. The synthesis relies on a stepwise addition in a 3′ to 5′-direction of individual 5′-*O*-dimethoxytrytil protected 2′-deoxynucleoside 3′-*O*-phosphoramidites (Figure 4A) until the desired DNA sequence is obtained. The synthesis is performed on a solid support, controlled pore glass (CPG), with the first nucleoside or a quencher (black hole quencher 1, BHQ-1) preloaded on the support (Figures 4B,C). The final stage in the synthesis of DNA probes is conjugation of fluorescein containing phosphoramidite (Figure 4D) at the 5′-end of the DNA sequence.

The overall yield of oligonucleotides varied from 1 to 16.4% for primers and <1% for probes with at least 80% purity as determined by denaturing PAGE (7 M urea). The yield, especially for probes, can be further improved by implementation of strict anhydrous conditions and probably longer coupling times during DNA synthesis as we noticed considerable amount of n−1 products during purification of the oligonucleotides. DNA probes contain large hydrophobic residues, BHQ-1 and fluorescein, which make purification of the final product difficult. We found that ion-exchange followed by reverse-phase HPLC purification used successfully for DNA primers did not result in isolation of pure DNA probes. Instead, purification of DNA probes was accomplished using preparative gel electrophoresis followed by extraction of the DNA from the gel using electroelution and final purification by reverse-phase HPLC. All DNA primers and probes were desalted and analyzed for their composition using electrospray ionization (ESI) mass-spectrometry (Table 1).

### Reverse Transcriptase Production and Function Testing

**Figure 5:**
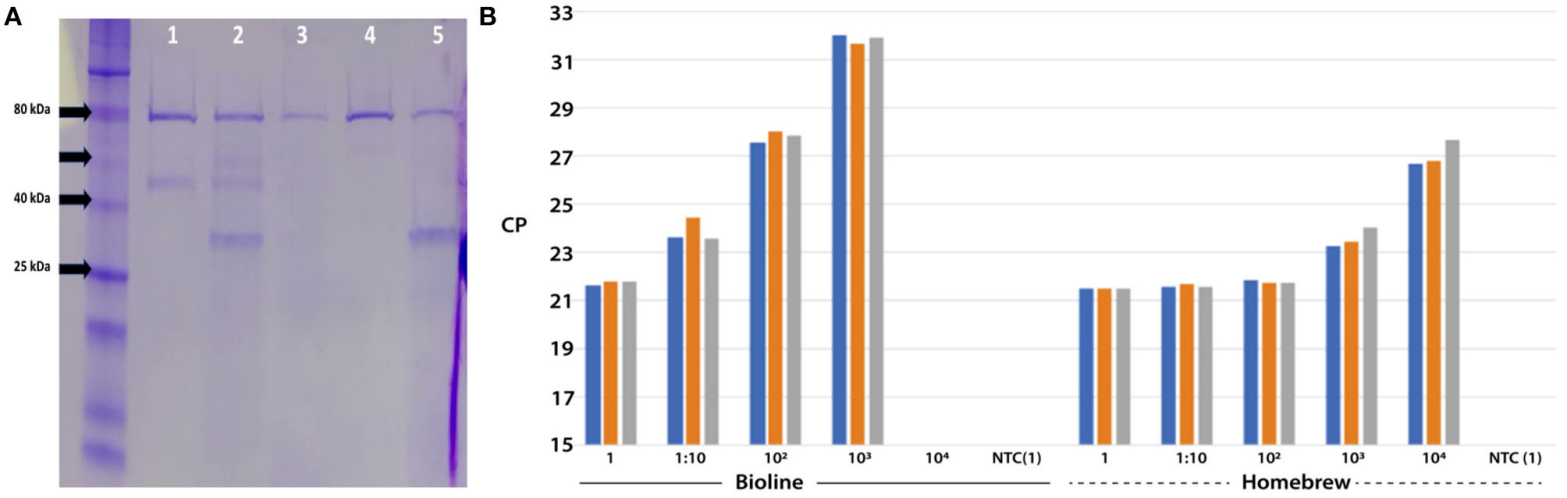
**(A)** SDS Page of Reverse Transcriptase enzyme: Lanes (1) *ab*TES RT (2) *ab*TES RT + protease digest (3) *ab*TES RT + digest with inactivated protease (4) MashUp RT (5) MashUp RT + protease digest; **(B)** Cp in triplicate for serial dilution of MashUp RT compared to BioLine RT.

A clone for a thermostable reverse transcriptase was a gift from A. Klenov (York University, Canada) and was referred to as “MashUP” RT. The clone was transformed into an *E. coli* expression host and grown to an OD_600_ of 0.8. Reverse transcriptase was then purified according to the method supplied. Protein purity was compared with two commercially-available reverse transcriptase enzymes. These were *ab*TES RT (AIT Biotech, Singapore; Figure 5) and Superscript III (Thermo Fisher; data not shown). MashUP RT presented as a single clean protein band that cut into two main fragments on protease digestion. Both commercial enzymes presented similar gel profiles (Figure 5A).

We used a modified method suggested by Vermeire et al. (10) to determine reverse transcriptase function in which a synthetic RNA for the SARS-CoV-2 E gene was substituted for MS2 RNA. RT-qPCR performance was compared to a commercially available one-step RT-qPCR system (BioLine SYBR No-Rox kit) by simply exchanging the commercial reverse transcriptase with the MashUP RT and performing a side-by-side comparison. A serial dilution of MashUP RT and the commercial RT was titrated against a constant 100,000 copies of the synthetic E gene RNA to measure performance (Figure 5B). MashUP RT performed well and appeared to have 100 × higher activity than the commercial product.

### *Taq* Polymerase Production and Functional Testing

**Figure 6:**
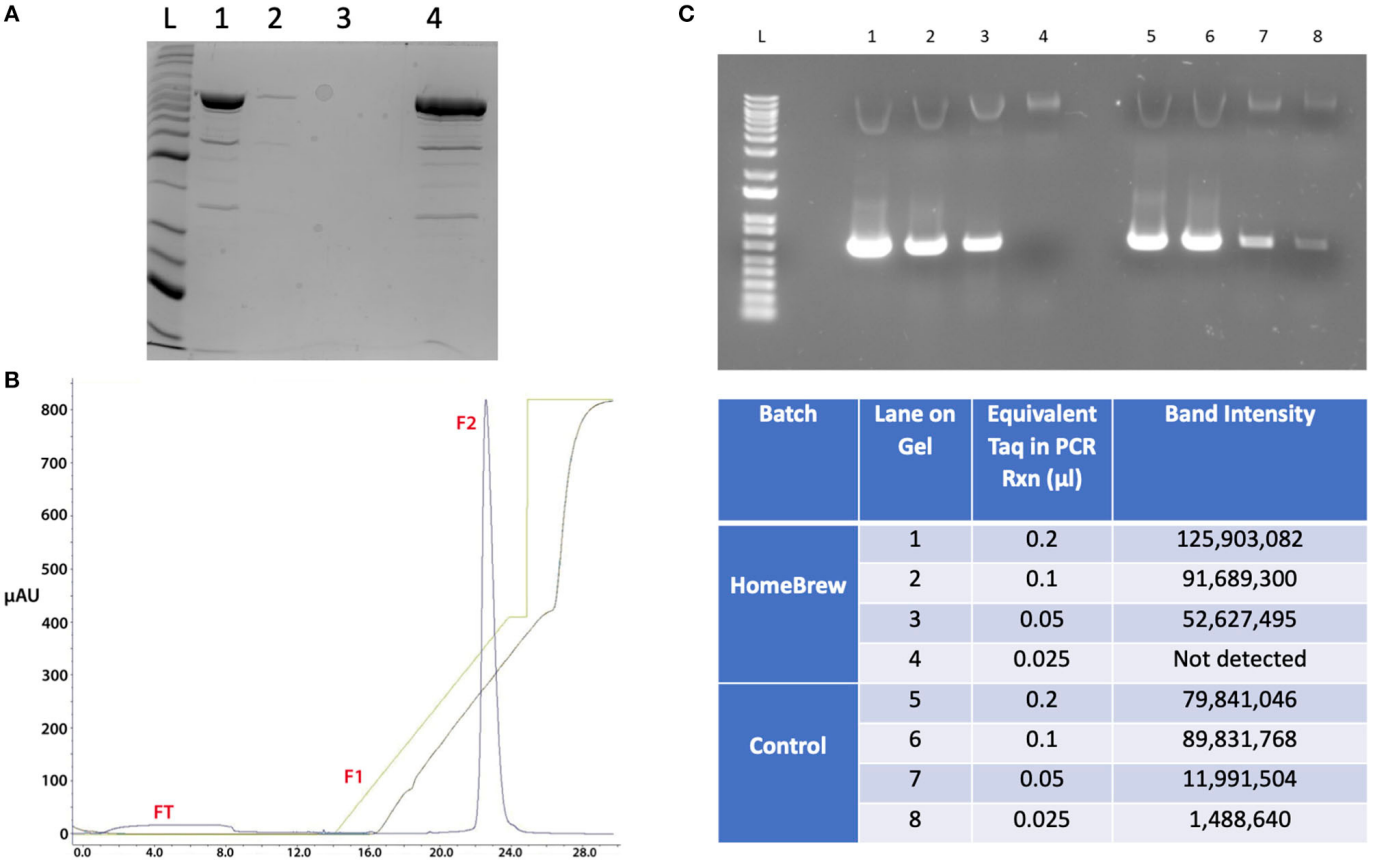
Purification and enzyme activity for HB Taq. **(A)** SDS Page showing 0.16 mg protein for MonoS fractionation prior to sample fractionation (Lane 1), the FT fraction (Lane 2), F1 (Lane 3) and F2 (Lane 4) fractions. **(B)** mAU readings for fractions FT, F1 and F2 from MonoS purification column. **(C)** Titration of HB Taq activity relative to a control enzyme using end-point PCR and gel image intensity: 0.2 μl enzyme (lane 1 and 5); 0.1 μl enzyme (lane 2 and 6); 0.05 μl enzyme (lane 3 and 7); 0.025 μl enzyme (lane 4 and 8).

A version of *Taq* polymerase cloned into *E. coli* DH5α was donated from the collection of J. Fraser (University of Auckland). Enzyme purity is given in Figure 6. This process produced a 67 ml solution of purified enzyme with an approximate protein content of 23 mg *Taq* polymerase. Total volume was made up to 200 ml by the addition of 133 ml Storage buffer (10 mM Hepes pH 7.0, 25 mM KCl, 0.1 mM EDTA, 1 mM DTT, 0.5% v/v Tween-20, 0.5% NP40, 50% glycerol). HomeBrew (HB) *Taq* was stored ready for use at −20°C in 40 ml aliquots.

Enzyme activity was tested against a well characterized batch of enzyme previously prepared from the *E. coli* DH5α::p*Taq* clone. Dilutions of the enzyme were used in an end-point PCR with limited temperature cycles to amplify a 659 bp cloned DNA fragment. PCR amplicons were fractionated over a 1% agarose gel and the fluorescence density measured from the image of each band (Figure 6). Comparison with commercial *Taq* polymerases and previous, well-characterized batches of in-house *Taq* were used to determine unit activity/μl. Comparison at 0.1 μl of stock *Taq* indicated both batches were similar, giving the new HB enzyme an activity of ~5 U/μl.

### One-Step Protocol From HB RT-PCR Reagents

As each individual component became available it was tested in both a two-step and a one-step RT-qPCR system by substituting the HB reagent for a commercial equivalent. Assays were performed using reference SARS-CoV-2 RNA extracted from virus originating and cultured from an infected NZ patient.

**Figure 7:**
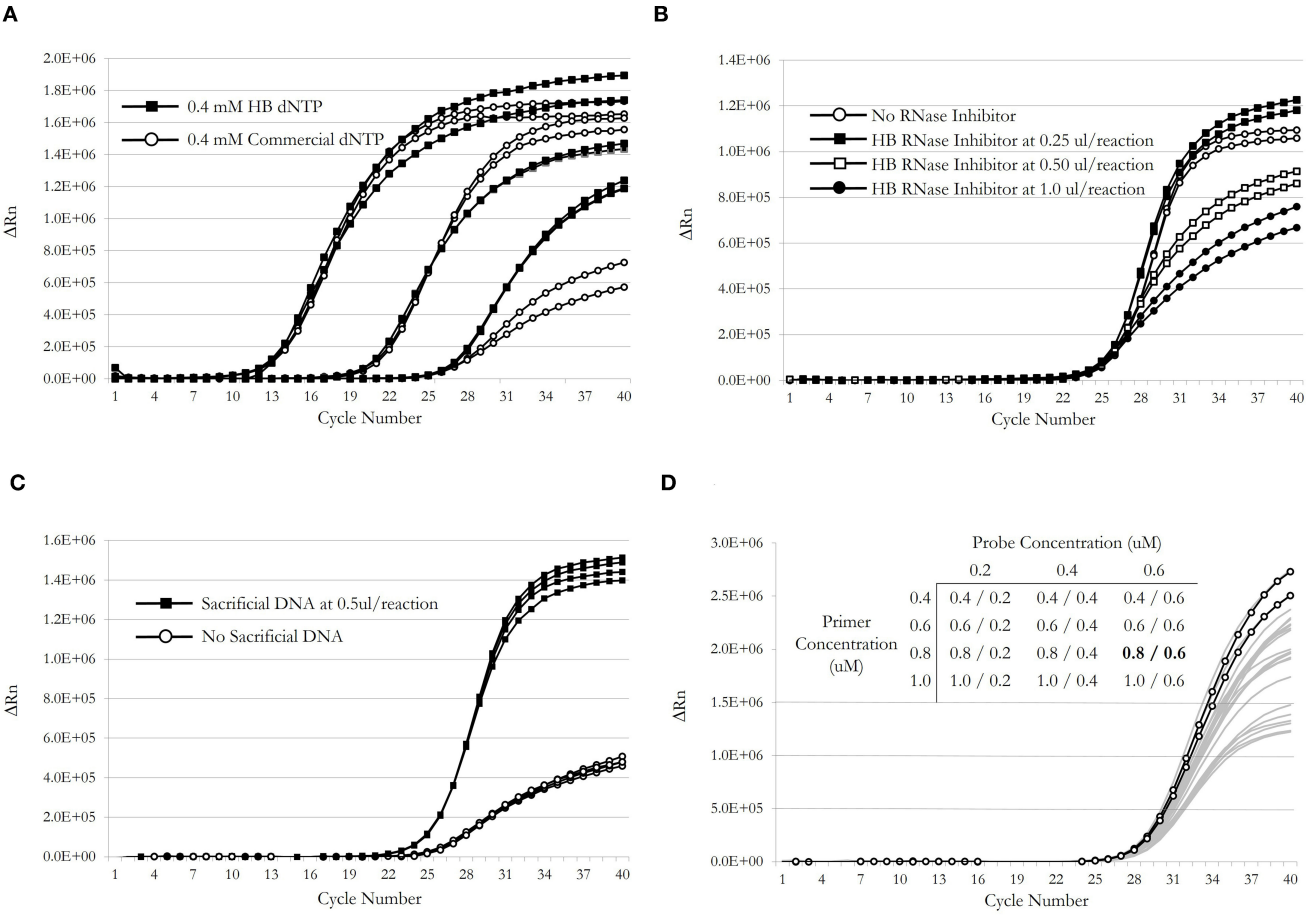
**(A)** PCR performance comparison of HB dNTP product relative to a commercially manufactured equivalent over three log dilutions of template. **(B)** Effect of inclusion of HB RNase Inhibitor product on SARS-CoV-2 RT-qPCR for CDC N-gene at varying concentrations. **(C)** Effect of adding sacrificial DNA to the RT-qPCR reaction mixture to mitigate non-specific and premature reporter moiety cleavage. **(D)** A primer/probe (CDC N-gene) concentration matrix was used to determine optimal reagent concentrations.

A buffered salt reaction solution consisting of 50 mM Tris.HCL (pH 8.4), 75 mM KCl was observed to be effective for both RT and PCR reactions performed in isolation and to provide satisfactory results when utilized in a one-step RT-qPCR format. A guiding principle for this effort was to minimize the number of components and where possible to exclude additional, but non-critical, components reported to enhance nucleic acid amplification assays (such as betaine, BSA, DMSO, trehalose, formamide, glycerol or detergents), rather than attempting to augment or complicate the mix with optional enhancer compounds. Uniquely, the HB reaction mix could be tailored to suit its one particular amplification task rather than aiming for the broader applicability and stability required of commercial mixes. Similarly, an endogenous normalization dye to correct for well-to-well optical variation (such as ROX or fluorescein) was considered non-essential. Final optimized working concentrations for HB primers and hydrolysis probe (CDC N-gene) were established at 0.8 and 0.6 μM, respectively (Figure 7D). A final concentration of MgCl_2_ of 2.5 mM in the reaction mix demonstrated optimal performance in this system, with HB dNTPs at 0.4 mM. HB RNase inhibitor was included at a final concentration of 0.25 μl per 20 μl reaction (Figure 7B).

During one-step RT-qPCR, high initial fluorescence values were observed at post-RT and pre-PCR stages, resulting in reduced ΔRn values after 40 cycles of amplification, relative to commercially prepared mixes. A series of exclusion experiments determined that this rogue fluorescence was originating solely from the labeled hydrolysis probe, as might be expected, and was likely a result of premature and non-specific cleavage of the reporter dye during reverse transcription. It was assumed, although not empirically determined, that nucleases co-purified with the MashUP RT or HB *Taq* enzymes, alongside 5′-3′ exonuclease activity arising from the native and non-“hot-start” HB *Taq* Pol I DNA polymerase during the reverse transcription step, may have been contributing to the premature cleavage of the reporter dye. The inclusion of exogenous or “sacrificial” DNA in the RT-qPCR reaction mix (in the form of genomic DNA purified from muscle tissue of the domestic fowl *Gallus gallus*), to a final concentration in the reaction of 25 ng/μl was observed to greatly alleviate, although not completely prevent, non-specific probe cleavage and high starting fluorescence values pre-PCR in the one-step format, most likely by providing a sacrificial substrate for contaminating nucleases (Figure 7C).

**Figure 8:**
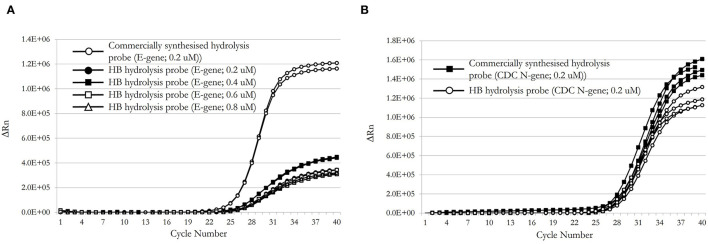
PCR performance comparison of HB E-gene **(A)** and HB CDC N-gene **(B)** with a commercially synthesized hydrolysis probes.

Desalted oligonucleotide primers and probes were reconstituted in TE buffer (10 mM Tris-HCl, pH 8.5, 0.1 mM EDTA) to 10 μM on receipt. In comparison to their commercially synthesized equivalent, the HB dual-labeled hydrolysis probes performed well in RT-qPCR although the HB E-gene probe exhibited notably lower peak fluorescence and higher Cq values than did the commercial E-gene probe; this was not improved by inclusion of higher probe concentrations in the reaction (Figure 8A). The CDC N-gene HB hydrolysis probe demonstrated equivalent performance to its commercially-synthesized counterpart (Figure 8B).

A finalized RT-qPCR reaction for CDC N-gene comprised 10 μl of 2 × PCR mix, 2 μl of 10 × enzyme mix, 1 μl of extracted RNA sample and 7 μl of nuclease-free water in a 20 μl total RT-qPCR volume. Cycling parameters comprised an initial reverse transcription step at 50°C for 20 min followed by a denaturation step at 95°C for 5 min and 40 cycles of qPCR comprising 95°C for 15 s and 60°C for 1 min.

### RNase Inhibitor Performance in HB RT-PCR

**Figure 9:**
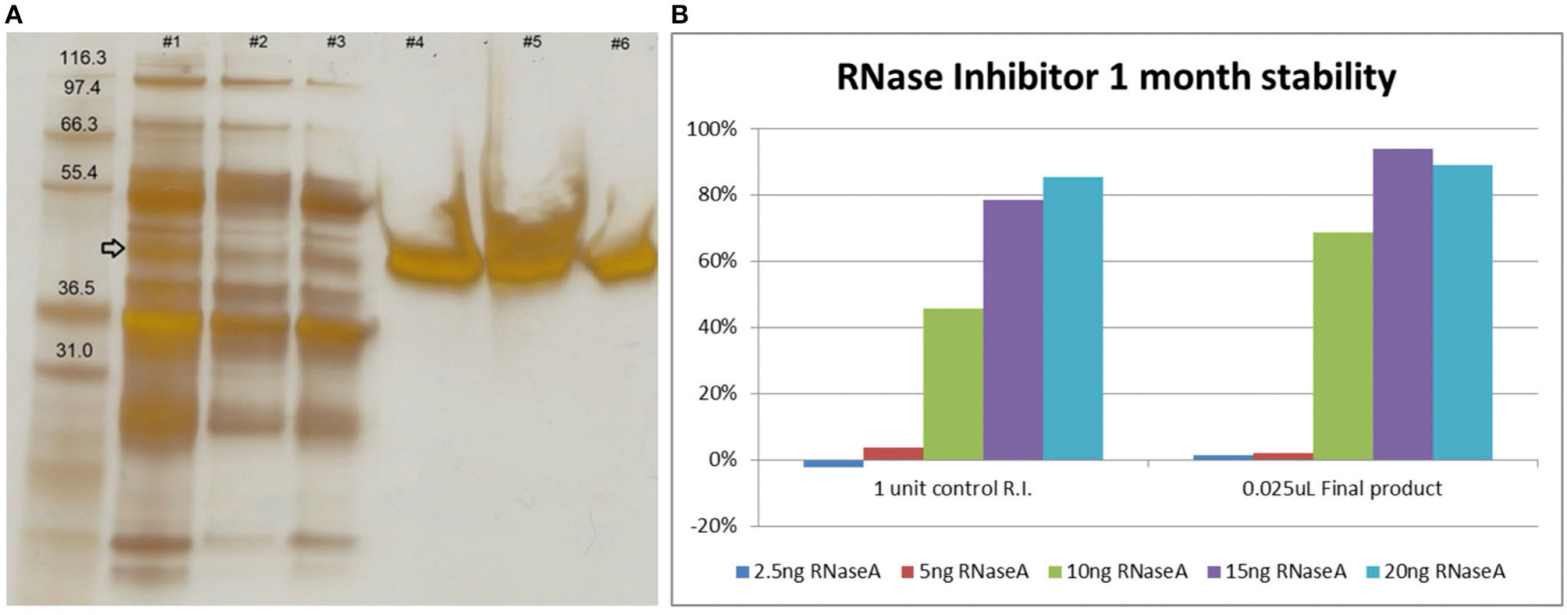
Quality control assay for RNase inhibitor, silver staining (~2 μg protein per lane) of SDS-PAGE gel **(A)** and RNase inhibitor assay (showing % activity of RnaseA) of final product 1 month after purification **(B)**. Lane 1: 1st re-suspended isoelectric precipitate with arrow indicating possible RNase inhibitor. Lane 2: 2nd re-suspended isoelectric precipitate. Lane 3: post-Sepharose binding sample. Possibly not all RNase inhibitor was captured. Lane 4: main peak of elution fraction 10–15. Lane 5: tail of elution (fraction 16–30). Lane 6: final product concentrated from the main fractions. Commercial RNase Inhibitor (Roche) was used as a positive control for the RNase inhibitor assay. By definition one unit of RNase inhibitor inhibits 5 ng of RNaseA activity by around 50%. In our case the amount of RNase A for the assay may have been overestimated.

The current protocol for RNase inhibitor could potentially purify 100,000 units (around 1 mg) per kg of sheep liver, with one single 40–50,000 Dalton band in SDS-PAGE gel detected using silver staining (Figure 9). Burton and Fucci (21) and Shapiro (22) suggested yields of 3–8 mg/kg are technically feasible. More optimisation would be required for routine production with centrifugation being one bottleneck in the process described here. Also, the yeast ribosomal RNA precipitation end-point assay has only a limited linear response to the amount of RNase inhibitor present, resulting in a semi-quantitative estimate of unit activity. A spectrophotometric cCMP assay would be more suitable for product release testing (21). Overall, however, purified sheep ribonuclease inhibitor remained stable after 1 month in −20°C storage when tested.

An evaluation of whether the HB RNase inhibitor improved overall performance of the HB RT-qPCR mix showed at high concentration it reduced DeltaRn target amplification, but, at 0.25 μl input (<10 U) results suggested the HB RNase inhibitor improved outcomes (Figure 7B). In summary, HB RNase inhibitor improved outcomes at low concentration while high concentrations led to unwanted inhibition of HB RT-qPCR. Given this observation HB RNase inhibitor was included in the HB RT-qPCR for clinical evaluation at low concentration.

### Clinical Evaluation

**Figure 10:**
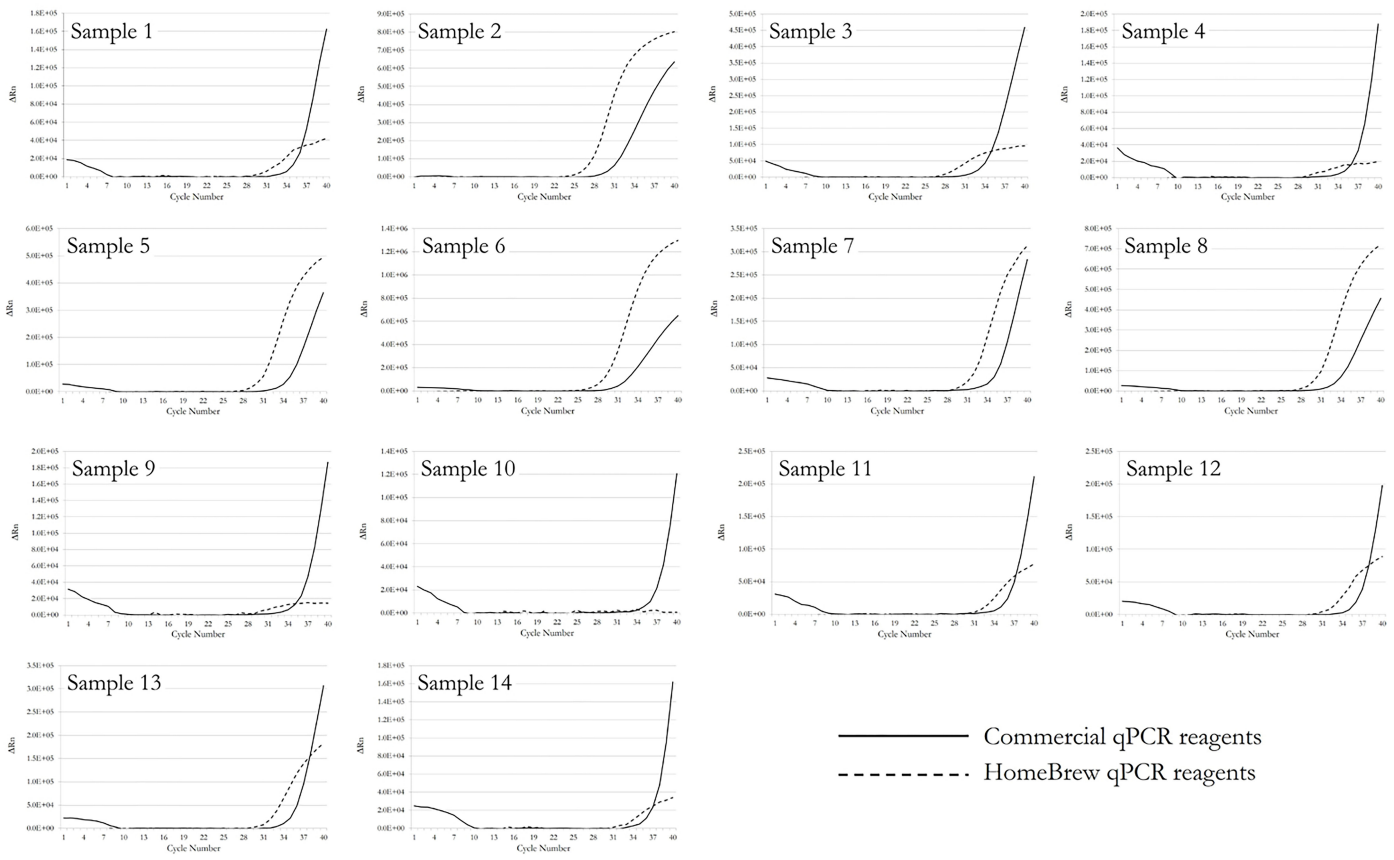
Comparative amplification curves for 14 clinical samples using the Quanta commercial RT-qPCR reagents and the HB RT-qPCR kit to detect the SARS-CoV-2 N gene.

Fourteen known positive clinical samples were compared using the HB RT-qPCR assay for N-gene and the standard assay used by ESR, a national reference laboratory for COVID-19 screening. The ESR assay used 15 μl of Quanta XLT mastermix with primers and probes synthesized by Biosearch Ltd. The sequences for the Biosearch primers and probe were the same as those used to synthesize the HB primers and probe. Results are given in Figure 10. Five microlitres of purified clinical sample RNA was used in each reaction and 40 amplification cycles were performed. In 13 cases HB RT-qPCR performance was comparable to the commercial product. However, there was one discordant call for sample 10 with HB calling this sample negative and the commercial product registering a positive, all be it at a late Cq. This points to HB RT-qPCR requiring further optimisation but confirms that it would be worth pursuing if NZ was presented with a doomsday scenario where reagent supply became restricted or unobtainable.

### How “Local” Is HB RT-qPCR?

Analysis of the methods presented here indicate that, at some point in the process, imported components have been used to synthesize HB RT-qPCR reagents. By working back through the methods from the end product, one finds a point at which a core, precursor material or important preparation tool was supplied by an overseas company. Examples are given in Table 2. A closer look at these items reveals that many could be synthesized in NZ if the need arose. For example, 2-deoxyribose and silylated nucleobases could be obtained through utilizing alternative processing methods by the food industry. Other components are raw materials used for a broad range of applications and industries in NZ, not just molecular biology, and will be present onshore in bulk as inventory in standard laboratories and processing plants.

**Table 2:** Examples of components used the are not sourced locally.

**Reagent/Equipment**	**Process**	**Source**	**Could reagent/equipment be sourced/synthesized in NZ?**
Nucleosides	Synthesis of dNTPs and nucleoside phosphoramidites for DNA synthesis	AK Scientific, Inc., Biosynth Carbosynth	Yes, from 2-deoxyribose and silylated nucleobases sourced from off-shore. Nucleosides could alternatively be isolated from natural sources (food industry)
SYBR Green	qPCR	Molecular biology providers: e.g. ThermoFisher (US), Biotium (US).	Yes. Would require further investment in scale up and capacity building
Fluorophores and Quenchers	qPCR	Lumiprobe, Genterra etc.	Yes, using published protocols and commercially available reagents.
Protein purification columns	Enzyme purification	Phenyl and MonoS columns are sourced off-shore through local suppliers.	Columns can be regenerated/reused.
Protein purification columns	RNase inhibitor	CnBr Sepharose (Cytiva), RNase A (Sigma)	Initial attempts to synthesize CnBr activated Sepharose were hampered by hazardous material shipping delays for CnBr.
Plasticware and Consumables	qPCR	Multiple suppliers: Eppendorf, Axygen, etc	Plates and optically clear adhesive seals could be made onshore. Injection molding services for the medical industry are available in NZ
Molecular grade water	All processes	Multiple suppliers: Roche, Millipore, Thermo Fisher Scientific	Water purification for use in RT-qPCR can be established in NZ using water purification systems currently located onshore and implementing a system of validation.

## Discussion

The goal of this work was to determine if NZ possessed the necessary expertise and infrastructure to produce the critical components needed for COVID-19 screening if access to supply was cut off for any reason. We have demonstrated that it does through the development of an effective RT-qPCR screening assay: HB RT-qPCR.

Our next question was whether onshore production capacity could service NZ's COVID-19 screening requirements. Under non-outbreak conditions NZ's rolling 7 day average is 5,000 tests per day (23). Our initial calculations and subsequent production volumes showed NZ HB RT-qPCR reagent manufacture could meet these demands. However, experience from the current August 2021 outbreak shows daily tests peaking in the tens of thousands. Servicing this high level of demand would be feasible, although necessary ramping up to larger scale production would take time to implement. For example, it would take ~1 month to scale up dNTP supply. In addition, HB RT-qPCR reagents in their current form do not meet international accreditation requirements and ideally would undergo a rigorous validation process to obtain clinical approval before being implemented.

Rapid scale up points to a number of issues. Namely, for immediate uptake, it is too late to source and set up production systems once doomsday has arrived. Therefore it is important to plan and test early so as to be prepared. The HB RT-qPCR exercise also highlights the downside of a globalized market. Though globalization under business-as-usual circumstances provides efficiencies and economies of scale, it also leads to a loss of local production capacity and a dependence upon overseas providers that may not be able to provide reagents under a global shutdown or extremely high demand. Such a situation was clearly demonstrated by COPAN's inability to supply the global demand for nasopharyngeal swabs during the early phase of the COVID-19 outbreak in 2020 (24). This led to a number of jurisdictions exploring in-house manufacturing options (e.g., Virginia, USA, Prof Melinda Poulter personal communication; ZenTech Medical, NZ) or evaluating alternative swab materials (25).

The issue of timing, regulatory approval and test volumes point to HB RT-qPCR not being available as an early response option for the COVID-19 pandemic but it does suggest that establishing an onshore, validated and tested pathway to production of molecular biological reagents for use in human testing can and should be established in case of future supply chain failures or in anticipation of new pandemic threats. We do not advocate that homegrown solutions should replace a globalized market but the lesson of prudence should be taken to heart. Without some capacity to provide a domestic-based solution, the ability to screen one's own population, by extension, is in the hands of foreign decision makers, overseas companies, their ability to supply a demanding market, and global transport systems.

Overseas sourced consumables had to be used at some point for every component of HB reagent synthesis. Our processes used less refined substrates and purification tools sourced from multinational suppliers. By setting up HB workflows these components have been identified. In many cases methods exist to produce even these components onshore and could be established if needed. Alternatively, as precursors are less refined and many are generically used across different industries, supply chains suffer less demand pressure and product can be sourced from multiple suppliers. These reagents could also be sourced from extant NZ chemical stores servicing other industries.

HB RT-qPCR required two enzymes: RT and *Taq* polymerase. For this work enzyme preparation was performed using research laboratory systems in keeping with a simple objective to generate functional product. Additional gains in purity, quality and quantity could be achieved through engaging with dedicated enzyme production facilities now that proof-of-principle has been established. NZ has these facilities, for example at SCION, that use large scale fermentation systems to produce large quantities of enzyme. Our proof-of-concept work using the MashUp and p*Taq* plasmids to produce viable enzymes for use in COVID-19 screening suggest that engaging with a local large scale producer would be a logical next step. A similar approach for producing RNase inhibitor could be undertaken.

RNA extraction from nasopharyngeal swabs was not specifically trialed as part of the HB initiative although supply of RNA extraction reagents was a critical concern at the beginning of the pandemic. The BOMB.bio protocol for making magnetic RNA extraction beads from easily obtained laboratory materials (8) has proved successful for COVID-19 screening at centers both in NZ and the UK and as part of modified diagnostic systems for viral testing [e.g., (26, 27)]. Use of BOMB.bio for RNA extraction from clinical samples meets the goals of self-sufficiency for NZ onshore production in case traditional supplies of RNA extraction reagent become unavailable.

Two pertinent issues have not been specifically addressed in this work: one is the cost of onshore HB reagent synthesis and the other is the issue of intellectual property (IP) ownership rights. Once synthesized the cost of HB materials is low. For example, the cost of HB dNTPs is estimated at NZ$0.30 per test but this price estimate does not represent labor or the costs of developing and further refining dNTP synthesis. Obtaining reagents more cheaply was not the focus of this work but rather understanding a potential response to a national crisis. Similarly, IP issues were not a concern of this work. In a national crisis NZ law permits the requisition of IP for the national good. Under S185 of the Patents Act 2013, the Crown may exploit an invention when a state of emergency has been declared under the Civil Defense Emergency Management Act 2002.

The objective of this study first and foremost was to reveal NZ's expertise and existing infrastructure to produce an assay that was fit for purpose, i.e., “good enough” to screen specifically for SARS-CoV-2 infection status if the situation demanded and if commercial reagent supplies were unavailable. We did not set out to create a HB competitor to commercially prepared reagents nor aim to fully develop HB such that it could not further benefit from additional development in terms of sensitivity, efficiency or stability with continued optimisation or addition of enhancer compounds. In reality, HB RT-qPCR displayed comparable performance to a commercial product. From this perspective we have surpassed expectations. Though NZ never completely ran out of reagents for COVID-19 testing, supply lines came close to being unable to deliver. Through this work we have demonstrated that NZ has both the expertise and, with sufficient lead time and forward planning, can build infrastructure capacity to meet this situation if it were ever to occur.

## Data Availability Statement

The original contributions presented in the study are included in the article/supplementary material, further inquiries can be directed to the corresponding author.

## Ethics Statement

Ethics approval and written informed consent were not required for this study in accordance with local legislation and national guidelines.

## Author Contributions

J-AS: wrote/coordinated first overall draft of the manuscript, coordinated reagent synthesis teams, and devised the reagent calculator. RO'B: end-to-end RT-qPCR protocol. RJH, AC, and HH: reagent testing and reagent validation. LJ: end-to-end RT-qPCR system clinical validation and oligonucleotide sequence recommendation. PM: reverse transcriptase production. JT: reverse transcriptase testing. JF: Taq production and wrote Taq production method. FC and LC: Taq production and testing. HK: probe and primer production. YS and VF: probe and primer production and wrote oligonucleotide synthesis method. WR: RNase inhibitor production. LL: RNase inhibitor production and wrote RNase inhibitor method. PR, LH, JW, and TS: dNTP synthesis. JU and JG: clinical advice, reagent sourcing, and oligonucleotide sequence recommendation. TH: BOMB.bio. TM: BOMB.bio reagent production. RH, BL, and MQ-M: SARS-CoV-2 reference material. PC: SDS page for RT. AK: developed the RT clone. RB: project initiator. All authors contributed to the article and approved the submitted version.

## Funding

This work was supported by the NZ Ministry of Health, the University Otago, the University of Auckland and the generosity of the NZ Science Community.

## Conflict of Interest

Authors JU and JG are employed by Southern Community Laboratories, Dunedin, New Zealand. Authors WR and LL are employed by South Pacific Sera, Washdyke, Timaru, New Zealand. Authors RO'B and PC are employed by MicroGEM NZ Ltd., 201 Princes Street, Dunedin, New Zealand. The remaining authors declare that the research was conducted in the absence of any commercial or financial relationships that could be construed as a potential conflict of interest.

## Publisher's Note

All claims expressed in this article are solely those of the authors and do not necessarily represent those of their affiliated organizations, or those of the publisher, the editors and the reviewers. Any product that may be evaluated in this article, or claim that may be made by its manufacturer, is not guaranteed or endorsed by the publisher.
